# The transatlantic fight against sickle cell disease: an interview with Marie Ojiambo

**DOI:** 10.1242/dmm.050629

**Published:** 2023-12-18

**Authors:** Marie T. Ojiambo

**Affiliations:** Charles River Laboratories, Safety Assessment, 905 Sheehy Drive, Horsham, PA 19044, USA

Sickle cell disease (SCD) is a debilitating disease that affects 120 million people worldwide, 66% of whom live in Africa [World Health Organisation (WHO) Regional Office for Africa]. In Africa, approximately 1000 children are born with SCD every day, and more than half of these children will die before five years of age (WHO Regional Office for Africa). A significantly smaller number of people with SCD reside in the USA – approximately 100,000 people – but this disproportionately affects people in the African American population, with SCD occurring in one out of every 365 births (Centre for Disease Control and Prevention). SCD is caused by a mutation in the β-globin gene (*HBB*), resulting in structurally abnormal haemoglobin that distorts red blood cells into a characteristically sickled shape. These rigid red blood cells cause blockages in blood vessels, leading to painful and recurrent episodes of vaso-occlusion in which tissues are deprived of oxygen, culminating in organ damage and reduced life expectancy. The most common treatment for SCD is hydroxyurea, which increases levels of a healthy form of haemoglobin (foetal haemoglobin) and decreases inflammation, but this treatment has severe side effects. Other treatments include folate administration and blood transfusions to combat anaemia, and, for some people with a matched donor, bone marrow transplants can offer a cure. More recently, breakthroughs have been made in the development of gene therapies to treat SCD that either repair the mutation in the adult β-globin gene or reactivate the production of healthy foetal haemoglobin (Wonkam, 2023).

Marie Ojiambo is a powerful advocate for people with SCD both in Kenya and in the USA. In Kenya, she founded the Sickle Strong Initiative (Box 1), and in the USA, she served as a consultant for Sickle Cell Disease Association of America (Box 1) and is a member of the oneSCDvoice working group (Box 1). Alongside her advocacy efforts, Marie is a Senior Scientific Associate at Charles River Laboratories, where she specialises in pre-clinical pharmaceutical research. As she was diagnosed with SCD as a child, she combines her lived experience with her extensive scientific expertise to bridge the gap between people living with SCD and researchers searching for a cure. In this interview, she discusses her evolving journey as a scientist, advocate and patient, and encourages a more international and collaborative effort to combat SCD.

**Figure:**
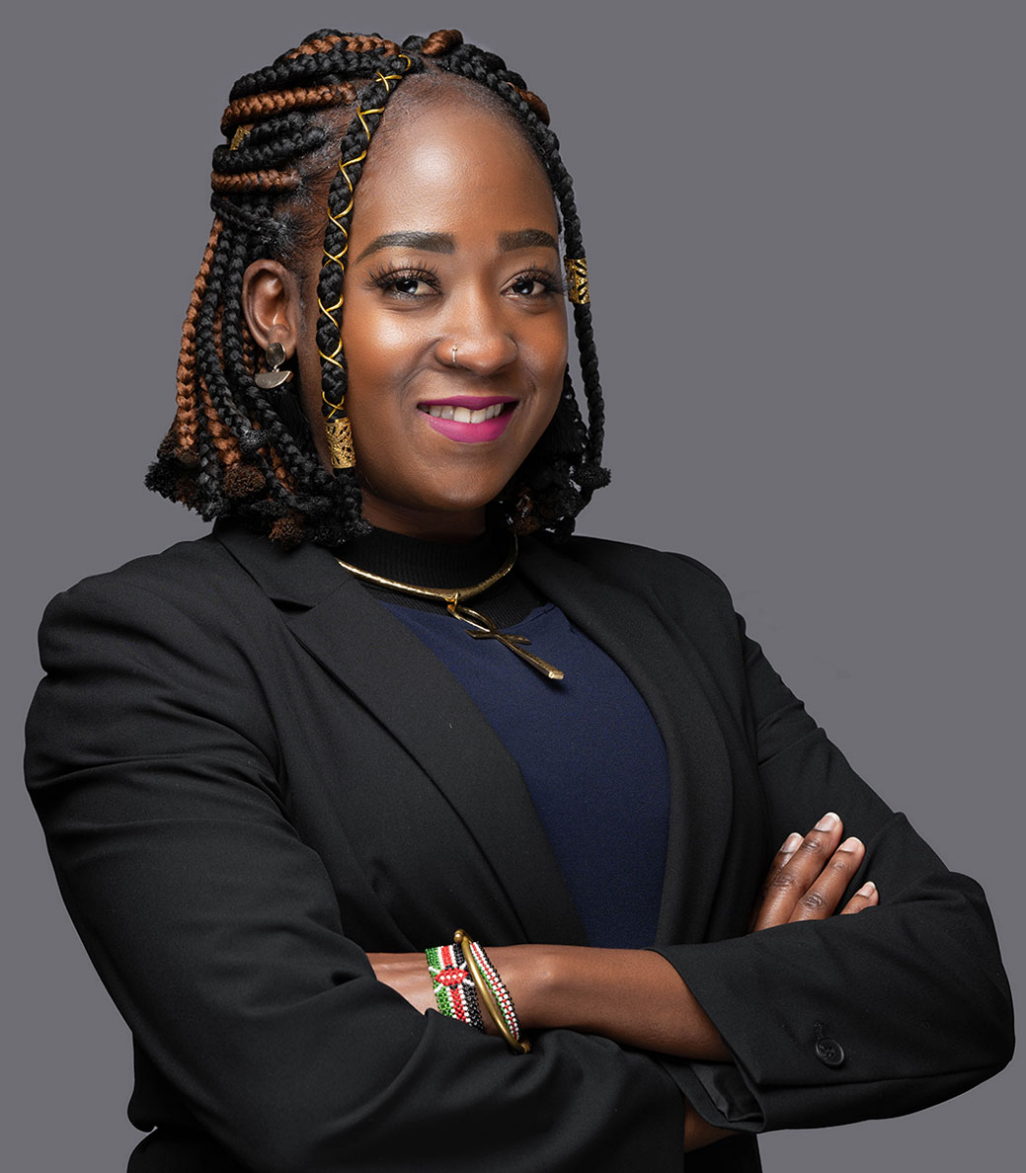
Marie Ojiambo

**Figure:**
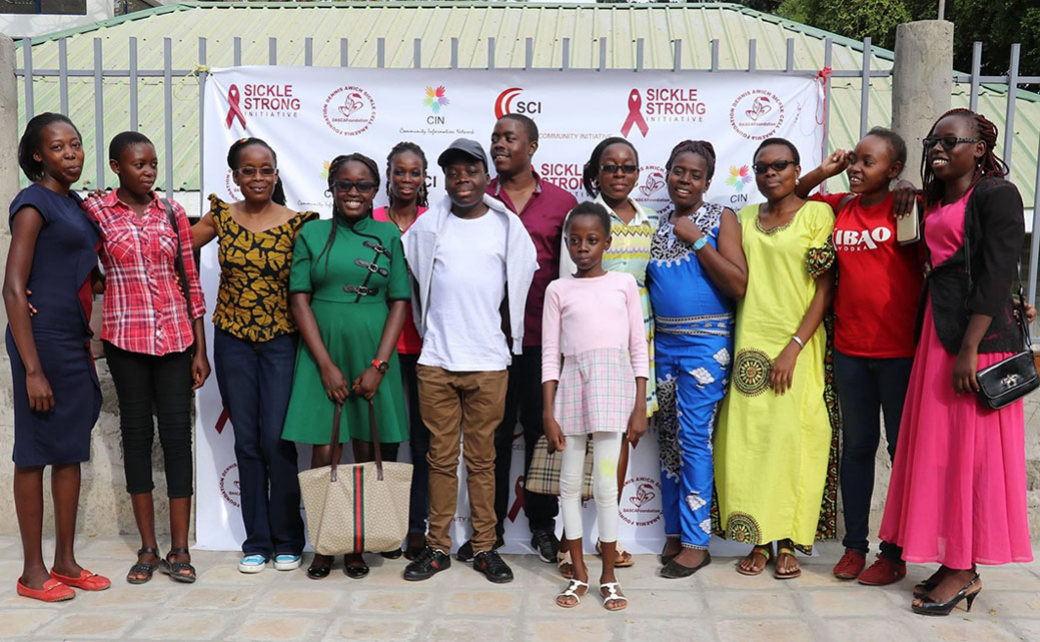
**Attendees of the Ongea Sickle Cell Awareness forum held in Homa Bay, Kenya in 2018.** This image is courtesy of Marie Ojiambo and is published under the CC-BY 4.0 license for this article.

**Box 1. Sickle Strong Initiative, Sickle Cell Disease Association of America and oneSCDvoice**

Sickle Strong Initiative (SSI) is a non-governmental organization based in Kenya that aims to action meaningful change in healthcare and education for people living with SCD. SSI raises awareness around the management, control and treatment of SCD. They also provide practical and emotional support for patients, their families and carers, by organising support groups and finding financial solutions to help patients access effective treatments and care. SSI engages with and challenges the government, pharmaceutical companies, insurance companies and other stakeholders to improve funding and support for people living with SCD in Kenya, and empowers them and their families to get involved with these advocacy activities.

The Sickle Cell Disease Association of America (SCDAA) is similarly focused on improving the quality of life of people living with SCD and raising public awareness around the disease. SCDAA brings together several organisations within the USA and beyond, having partnered with the government, private organisations and not-for-profit agencies, including the National Institutes of Health and the Centers for Disease Control and Prevention. SCDAA has six core areas of focus: Research, Public Health Education, Professional Health Education, Patient Services, Community Services, and Support to Global Organizations and Practitioners. In terms of research, SCDAA promotes basic and translational research to expand our understanding of the pathogenesis of SCD, with the ultimate goal of developing effective treatments. They advocate for increased and coordinated efforts from the government to support SCD research and to enact informed decisions in policy. Furthermore, the annual SCDAA conference provides a platform to present and discuss the latest advances in basic and clinical research. They also provide information for people who wish to participate in research, via initiatives such as the Clinical Trial Finder. SCDAA also facilitates a range of educational and practical support mechanisms for patients by partnering with dedicated medical centres and organisations that provide genetic testing and counselling, insurance counselling, financial aid, pharmacy assistance and psychosocial support for patients and families affected by SCD. Finally, SCDAA assists advocacy organisations and health practitioners globally, with particular focus on countries in Africa to help improve SCD care.

oneSCDvoice is in partnership with SCDAA and is also based in the USA. Its aim is to provide a space for people with SCD to find relevant news and knowledge and to make connections with the community. They provide trusted resources and educational tools to help patients and their families learn more about SCD and access support. Furthermore, patients, their families and advocates can access the ‘Social Wall’ to chat and connect with each other.

## What inspired you to study and work in pharmaceutical research?

When I finished high school, I initially wanted to become a medical doctor. My mother is a medical doctor by training, so I talked to her about it, and I thought I could give it a try. I did my undergraduate degree in Kenya at the University of Nairobi, and in my first year of training, I studied anatomy, pathology, microbiology, and so on, which was standard, but seeing very sick people during my senior clinical rotations in the ward wasn't easy for me. I found myself to be a very empathetic person. I discussed it with my mother, and she suggested I try to go into research and that the first step could be to go to the School of Pharmacy at the University of Nairobi. I wasn't really excited about the possibility of ending up behind the counter dispensing drugs, but I realised that there were various tracks in pharmacy. The first three years involved a lot of reading about pharmaceutical science, but in the fourth year, we had rotations outside of the school setting. We had a hospital rotation referred to as Clinical Pharmacy, where you do ward rounds with doctors and participate in therapeutic drug monitoring. We also had an industrial rotation where you had an opportunity to work in a pharmaceutical company and see the process of drug discovery and/or development from the bench to the bedside. I completely fell in love with the industrial rotation.

When I was finishing up my undergraduate degree at the School of Pharmacy, my family relocated to the USA. When I joined my family in the USA, I pursued a master's degree in Industrial and Physical Pharmacy at St. John's University College of Pharmacy and Health Sciences in New York. This was mainly formulation science, where we took an active compound and combined it with inactive compounds to develop a stable final drug product. This product would then be investigated in a pre-clinical model before it moved to the clinic. Once I graduated, I was recruited as a Formulations Research Associate at Charles River Laboratories. I have since grown in my roles and I am currently working as a Senior Scientific Associate and Study Director within the company. As a Study Director, I act as the single point of control for studies and ensure that the projects assigned to me are managed effectively and in accordance with good laboratory practices to confirm that the drugs are safe and efficacious for the patients that need them. My specific role focuses on reproductive toxicology, so we're looking at how drugs affect the unborn foetus and follow that into the juvenile animal model. We want to know whether a drug would be safe for a paediatric population or for pregnant and nursing mothers.

## You have a wide range of roles beyond working in industry. How have these experiences benefitted each other and how does your work vary in Kenya compared to the USA?

A wise bird once told me that there are three realms of your professional life: when you find your purpose, when you're working in it, and when you can use that work to give back to society. I find that my career development, along with my opportunities to give back to society and to grow as a person, have been entwined with my experience of living with SCD.

I founded the Sickle Strong Initiative in Kenya in 2013 because the disease is more prevalent in Kenya, and the need is greater. SCD has historically been found in areas that are endemic for malaria. That is because having one allele with the genetic mutation that causes SCD confers a degree of resistance against malaria. I come from one of the regions that is endemic for malaria – the western region of Kenya – where almost 40% of the population lives with SCD or the trait. I began to advocate after I was sick in hospital in the USA. I saw a lack of education and misinformation in the way the nurses and doctors treated me. I wondered how this was for other patients who might not have the knowledge that I have, the healthcare background or have a parent who is a doctor. I was very fortunate that I could advocate for myself in a hospital setting. From the need to provide education and awareness to others living with the disease and the general public who interacted with these patients, Sickle Strong Initiative was birthed. I wanted to give a voice to those who are living with the disease, not only patients, but also parents of patients, who may not understand how to advocate for them. When I founded it, there was less of a voice in terms of advocacy in Africa, and not a lot of people had begun to speak about the condition and its debilitating effects. At the time, a lot of patients were scared to speak up because of the stigma that was associated with the disease; many patients in Kenya had lost their jobs when their employers found out that they have a chronic condition and may be more of a burden to the company than anything else. Also, a lot of people in Kenya do not understand what SCD is and it is still tabooed. In some regions, kids born with SCD into some families were considered bewitched, or the mother was considered bewitched. So, I aimed to educate the population to help them understand that it is not the mother's fault, that it arises from both parents and that hopefully we can reduce its prevalence through genetic counselling. Many people in my region of Kenya cannot afford therapeutic options, even as basic as hydroxyurea or folate, and so, my number one focus in terms of my advocacy efforts was getting these simple treatments to be more accessible. I reached out to healthcare organisations asking them to donate therapeutics.

Since then, my advocacy style has changed because when I started to advocate for patients, it was truly just me and a handful of people in Kenya. In the USA, SCD is considered a rare disease and a lot of advocates within the USA are much more vocal and proactive, which was wonderful to me. I realised at that time that there was an awakening of voices in the USA, as a lot of patient advocates, patient champions, clinicians and researchers decided to speak up about the condition. We experienced a surge in the research pipeline, as a lot of new therapeutics were being investigated. I since modified my advocacy style to align with my career. I wanted to use my scientific work to make a difference in the SCD space. I started to consult with various pharmaceutical companies, patient organisations and NGOs. I wanted to focus on gene therapies and explore how they could potentially provide a cure for diseases, such as SCD and other genetic conditions. Because I am a scientist, I can speak with and educate other scientists in this space. So now, I use my experience to edify the research questions to help researchers understand what is needed for patients.

I can talk to patients, researchers and clinicians in Kenya about what's going on in the research spaces in the USA […] and, on the other hand, I can go back to the USA and talk to the clinicians about what's going on in Africa.

The funding for research and the pharmaceutical companies and academic institutions that can do the research are mostly not found in Africa; they are found in countries where pharmaceutical research is more advanced. So, in terms of research opportunities, the USA is much further ahead compared to many low- and middle-income countries. Working in the USA has modified my advocacy approach, especially in Kenya. I can talk to patients, researchers and clinicians in Kenya about what's going on in the research spaces in the USA. I can tell them what cutting-edge technologies, such as gene therapies, are in the pipelines. And, on the other hand, I can go back to the USA and talk to the clinicians about what's going on in Africa, which is going to be one of the biggest markets for these emerging therapeutics. I can tell them what therapies are needed and what the patients need but cannot access. I can tell them about the quality of life of people with the disease, and what they'd like to see, as regards to the research questions. Although access to drugs in the United States is still a challenge, health insurance options are much better compared to Kenya. Also, therapies like blood transfusions are easier to come by in the United States. One life saving therapeutic that I received in the US was a red cell exchange transfusion, but in Kenya we probably have two or three hospitals that offer that type of life saving intervention to patients. So, there is a stark difference between the two in terms of availability of healthcare, advocacy and research opportunities.

## How does living with SCD impact your work?

I think it enriches my work because I can personally talk about the matter. Sometimes I sit at these discussions about therapeutics for SCD in pharmaceutical companies, and you find that a lot gets lost in the science. The companies are trying to understand the science, but they don't understand what the patient actually needs. It's funny that when researchers started to listen to patients more, they found out that, yes, the majority of patients would like a cure, but for those who are older and are not good candidates for, for instance, bone marrow transplant procedures, they want a way to manage the condition better. For me, managing my condition is about managing my fatigue, which is one of the biggest complications when it comes to living with this disease. I don't want to wake up and feel like I've been running a marathon all night. Managing my complications is also about reducing the number of times I have to go into hospital because of vaso-occlusive episodes, which can be as often as three or four times a year for some people. Another patient might have issues with pain in a joint due to vascular necrosis. So, I think it's really important for clinicians and researchers to understand what the patients need. They understand the need to push for a cure, but sometimes I feel like it's important for them to also just sit down with different patients and ask, ‘What would you like for us to work on in the laboratory?’ Living with SCD has given me a voice to answer these questions for researchers.

Aside from that, being a healthcare practitioner has helped me in my patient advocacy journey because I am able to understand the pathophysiology of the disease and communicate that in layperson's terms to other patients. I think that has been the greatest win for me in my advocacy style, as that is also how I managed to speak with all these chief executive officers (CEOs) and consult with various organisations. They saw that as an opportunity because I could help them go out and speak to patients about their therapeutics. I am someone who suffers from the condition and can explain what their therapeutic will mean to other patients.

Clinicians or researchers in the USA should bring their knowledge to emerging markets, such as Africa, to teach how these therapeutics are developed and even establish pharmaceutical companies in these countries.

## Gene therapy for SCD is a breakthrough, but what research still needs to be done to ensure that this treatment can help people globally?

As SCD is a relatively rare genetic condition, a lot of these new therapeutics are quite expensive, because manipulating the human genome is not an easy feat. It's difficult, it's time consuming, it's expensive. Right now, we're in the clinical trial stage of gene therapies, but once gene therapies hit the market and are approved by the US Food and Drug Administration (FDA), hopefully, they will be made more accessible as we get to know more about them. As we get to learn how to upscale the treatment, we make it more accessible, not only in the USA or other high-income countries, but also in the emerging markets, such as African countries. That also means building the capability to manufacture these therapeutics in countries like Kenya. Clinicians or researchers in the USA should bring their knowledge to emerging markets, such as Africa, to teach how these therapeutics are developed and even establish pharmaceutical companies in these countries. We also need to have hospitals where it is possible to do such procedures in Africa, with clinicians that are trained to take a patient from the preparation stage of gene therapy right through to the end of the process. So just making the knowledge accessible, I think, is the biggest way that we can make these therapeutics accessible to emerging markets in Africa. It will take some time, and I do understand that the infrastructure is still growing, but I believe that it is possible. For instance, the first gene therapy procedure was done in Kenya a few months ago, in one of the big hospitals in Nairobi. So, these things are possible, but it will take time and a lot of finances to get there.

Unfortunately, in terms of accessibility right now, it's really the insurance companies that could help with this. A lot of insurance companies in Africa will not provide adequate insurance for people with SCD because it's a pre-existing condition. Anytime we host an event, such as the annual sickle cell forum, Ongea, in Kenya, I like to invite insurance companies, so that they know we are depending on them to get these therapeutics to the people that need them.

In Kenya, we are still trying to access the drugs that hit the market decades ago, so gene therapy still looks like a pipe dream. But hopefully, we will get there.

## What is your overall view of emerging treatments for SCD, such as gene therapy?

There is no bad therapeutic. Anything that is an option for the treatment, management or cure of the disease is something that is completely welcome. Before therapeutics such as hydroxyurea even hit the market, it had been close to 100 years without any treatment option for this patient population. Now, in Kenya, we are still trying to access the drugs that hit the market decades ago, so gene therapy still looks like a pipe dream. But hopefully, we will get there.

Gene therapies and stem cell transplants are obviously life-saving options, but for transplants, you have to go through a lot of procedures and receive high doses of chemotherapy to suppress your own bone marrow before you receive the transplant. And then there are other issues, such as graft versus host disease, potential infertility, or infection that may arise during the process. At the end of the day, it's wonderful if you are cured of the disease, but when you think about the side effects and the financial impact that it has… it's a lot. I've spoken to a lot of patients who have gone through bone marrow transplants, and one parent said that he would not advise it, even after his child was successfully treated. He said, ‘This is a therapeutic that I would never recommend, because it took such a toll on the family unit, psychologically and financially.’

I'd love for there to be a cure, but for me – speaking now as a patient – the number one priority would be a simple drug that increases the levels of healthy haemoglobin, without having the side effects of hydroxyurea.

I'd love for there to be a cure, but for me – speaking now as a patient – the number one priority would be a simple drug that increases the levels of healthy haemoglobin, without having the side effects of hydroxyurea. A lot of patients are non-compliant with hydroxyurea because of the side effects. And even a small increase in haemoglobin levels could help to treat fatigue, vaso-occlusive episodes and organ damage. If I had 30 billion dollars to throw into a pipeline, that's what I'd ask for.

Overall, I like to encourage research scientists to continue working on different therapeutic options. And I encourage researchers and clinicians to invite patients and advocates to discussions about these research pipelines and areas of focus. I think we are doing more of that and now I can see more of a patient-centred focus when it comes to researching therapeutics for the disease, which has been absolutely fantastic.

## Conclusions

Despite efforts to improve treatments and management, SCD still has devastating effects. In 2019, 38,403 deaths due to SCD were recorded in Africa, which is a 26% increase from 2000 (WHO Regional Office for Africa). As SCD is much more prevalent in populations originating from regions that are endemic for malaria, the disease has the highest burden in Africa and in populations with African ancestry. These populations not only have a higher genetic risk of SCD but face extreme challenges in accessing adequate healthcare. Marie Ojiambo's advocacy work, both in the USA and in Kenya, is ensuring that standard SCD treatments and care are more accessible to everyone.

Looking to the future, gene therapies for SCD may offer a cure. But will this just be a glimmer of hope for people living with SCD in low-income countries? And does everyone with SCD want to undergo the intense medical process of gene therapy, when a milder therapeutic would be sufficient for them to live a relatively healthy life? Marie makes it clear that researchers need to continue a broad line of investigations to build an artillery of treatments that serve the needs of the wide range of people with SCD. She also encourages researchers to include more patients in discussions revolved around research that will ultimately affect them. By working within a pharmaceutical company and consulting with several others, such as Pfizer, Marie can steer the conversation back to the people living with SCD and their needs. Her strong influence may even encourage some of this research to be developed within countries with the highest prevalence of SCD, which would be a promising step towards equitable healthcare.
